# BMP Signalling at the Crossroad of Liver Fibrosis and Regeneration

**DOI:** 10.3390/ijms19010039

**Published:** 2017-12-23

**Authors:** Blanca Herrera, Annalisa Addante, Aránzazu Sánchez

**Affiliations:** Department of Biochemistry and Molecular Biology, Faculty of Pharmacy, Complutense University of Madrid (UCM), Health Research Institute of the Hospital Clínico San Carlos (IdISSC), 28040 Madrid, Spain; blancamh@ucm.es (B.H.); annalisa.addante@gmail.com (A.A.)

**Keywords:** BMP, liver, fibrosis, regeneration, TGF-β, chronic liver disease

## Abstract

Bone Morphogenetic Proteins (BMPs) belong to the Transforming Growth Factor-β (TGF-β) family. Initially identified due to their ability to induce bone formation, they are now known to have multiple functions in a variety of tissues, being critical not only during development for tissue morphogenesis and organogenesis but also during adult tissue homeostasis. This review focus on the liver as a target tissue for BMPs actions, devoting most efforts to summarize our knowledge on their recently recognized and/or emerging roles on regulation of the liver regenerative response to various insults, either acute or chronic and their effects on development and progression of liver fibrosis in different pathological conditions. In an attempt to provide the basis for guiding research efforts in this field both the more solid and more controversial areas of research were highlighted.

## 1. Introduction to BMPs

Bone Morphogenetic Proteins (BMPs) are multifunctional cytokines that belong to the Transforming Growth Factor-β (TGF-β) superfamily, being in fact the largest TGF-β subfamily comprising more than 15 ligands in mammals. BMP ligands have been classified into different groups according to their sequence and structural homology: BMP2 and 4; BMP5, 6, 7, 8 and 8B; BMP9/Growth Differentiation Factor-2 (GDF)-2 and BMP10; BMP11/GDF 11 and GDF-8; BMP12/GDF-7, BMP13/GDF-6 and BMP14/GDF-5; BMP15/GDF-9b and GDF-9; GDF1 and 3; BMP3 and BMP3b/GDF-10. BMP1 is actually a metalloprotease and it is not considered a member of the family [1,2].

Ligands of the BMP family are synthesized as large precursors consisting in a N-terminal signal peptide that directs the precursor to the secretory pathway; a prodomain and a C-terminal peptide. Although there is some debate about where the processing takes place, endoplasmic reticulum (ER) or Golgi, the pro-domain is cleaved at a consensus Arg-X-X-Arg site by serine endoproteases to produce the mature protein, which is subsequently secreted. The pro-domain may or may not remain non-covalently associated to the mature peptide under physiological conditions playing an important regulatory role on ligand activity [3]. Most of mature-active BMP molecules are formed by bisulfide-linked monomers of the same BMP ligand, forming homodimers. However, both in vitro and in vivo evidence demonstrates the existence of several BMP heterodimers [4,5,6].

BMPs were originally discovered for their capacity to induce bone and cartilage formation and fracture repair and to regulate growth and differentiation of chondroblast and osteoblast cells in vitro [7,8]. Their role in early development is also well documented, including dorsal-ventral patterning, organogenesis and cell differentiation. Thus, BMPs act as mitogens and morphogens, being their expression tightly controlled in space and time during embryogenesis [9]. More recently, a role for BMPs in adult tissue homeostasis has been revealed. Evidence shows that BMPs regulate several cellular processes, including cell proliferation, differentiation, chemotaxis, apoptosis and migration in many different cell types and play critical roles in different organ systems, including skeletal, cardiovascular, pulmonary, urinary-reproductive and gastrointestinal systems [10]. Studies from transgenic and knockout mice, as well as animals and humans with naturally occurring mutations in BMPs and related genes have shown that deficiency of BMP production or function lead to abnormalities or severe diseases. Besides, deletion of various components of the BMP pathway is embryonically lethal.

## 2. BMP Signalling: Canonical and Non-Canonical Pathways

BMPs activate Smad-dependent (canonical) and multiple Smad-independent (non-canonical) signalling pathways to directly affect gene transcription. They initiate the signal transduction cascade by binding to cell surface receptors and forming a heterotetrameric complex comprised of two dimers of type I and type II serine/threonine kinase receptor [11,12,13].

### 2.1. BMP Receptors

BMP receptors (BMPRs) are serine/threonine kinase receptors, composed of three parts: a short extracellular domain with 10–12 cysteine residues, a single membrane-spanning domain and an intracellular domain with the active serine/threonine kinase region. The type I receptor carries two additional motifs, a glycine/serine-rich region preceding the kinase domain (GS-box) and a short region of eight amino acids, termed L45 loop, within its kinase domain. The type II receptor kinase is constitutively active [14,15]. The specificity of the intracellular signals is mainly determined by type I receptors. BMP ligand associates with the extracellular domains of BMP receptors at the cell surface to produce a signalling assembly. Specifically, the activation of type I receptor kinase requires ligand binding, ligand–receptor oligomerization and transphosphorylation of its GS-box via the type II receptor [16].

Five known BMP type I receptors are available for the 12 BMPs: the activin receptor-like kinase 1 (ACVRL1 or ALK1); type 1A activin receptor (ActR-1A or ALK2); BMP receptor type 1A (BMPR1A, also known as ALK3); the activin receptor type-1B (ACVR1B or ALK-4) and type 1B BMP receptor (BMPR-1B or ALK6) [11,12]. On the other side, a total of three type II receptors are known to interact with BMPs: type 2 BMP receptor (BMPR-2), type 2 activin receptor (ActR-2A) and type 2B activin receptor (ActR-2B). Only the BMPRII is specific for BMPs. The mechanism of the heterotetrameric signalling complex formation varies among ligands. As illustrative examples, while BMP6 and BMP7 interact with type II receptors and recruit type I receptors, BMP2 and BMP4 preferentially bind type I receptors and recruit type II receptors [13]. Diversity of BMP signalling responses is determined by different factors, among them, a cell type and context-specific expression and formation of different receptor combinations. Despite the fact that different type I and type II receptors present high level of homology, they are non-redundant. Further research is nonetheless necessary at this level.

### 2.2. Smad-Dependent Pathway

The Smad protein family can be divided into three subgroups including the receptor-regulated (R-Smad), common-mediator (co-Smad) and inhibitory Smads (I-Smad). The R-Smads include Smad1, Smad2, Smad3, Smad5 and Smad8/9; the co-Smad includes only Smad4, which interacts with R-Smads to participate in signalling; and finally, the I-Smad include Smad6 and Smad7, which negatively regulate Smads activation and regulate receptor stability [17,18].

**Figure 1:**
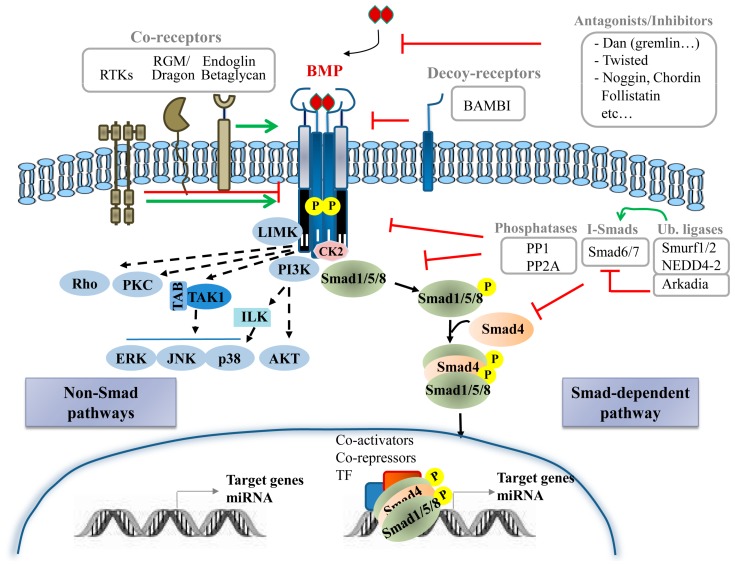
BMP-triggered signalling pathway and major regulators. BMP ligand binds to its membrane serine/threonine kinase receptors type I and type II. Once the heterotetrameric complex is formed, constitutively active type II receptor transphosphorylates the type I receptor to initiate intracellular signalling. In the canonical pathway, R-Smads are phosphorylated by type I receptor and subsequently bind to Co-Smad, Smad4. The resulting complex translocates to the nucleus where it recruits specific co-activators, co-repressors and transcription factors to regulate the expression of target genes and microRNA that in turn will target specific mRNAs. This pathway is strictly controlled by different means. Extracellular antagonists/inhibitors (Dan, Twisted, Noggin, etc.) and decoy receptors (Bambi) and co-receptors (endoglin, beta-glycan, RGM/dragon, RTKs…) in the plasma membrane regulate binding of BMP ligand to its membrane serine/threonine kinase receptors. Additional regulators include CK2, I-Smads, phosphatases (PP1 and PP2A) and ubiquitin ligases among others. BMP also activate several non-Smad pathways, including Rho small GTPases, LIMK, PI3K/AKT, PKC and ERK, JNK and p38 MAPKs which modulate BMP cellular responses. Bambi: BMP and activin membrane bound inhibitor; CK2: Casein kinase II; ILK: Integrin-linked kinase; LIM kinase: Lin-11/Isl-1/Mec-3 gene products kinase; MAPK: mitogen-activated protein kinase; PI3K phosphatidylinositol 3-kinase; PP1: Protein phosphatase 1; PP2A: Protein phosphatase 2A; RGM: repulsive guidance molecules; RTK: receptor tyrosine kinases; TAK: TGF-β-activated kinase; TAB: TAK binding proteins.

Upon formation of a heterotetrameric complex, the constitutively active type II receptor transphosphorylates the type I receptor at the GS domain. This activates the type I receptor and allows phosphorylation of the immediately downstream substrate proteins R-Smads at a C-terminal SSXS motif [19]. Of the eight Smad proteins identified in mammals, Smad1, Smad5 and Smad8 are R-Smads activated without selectivity by BMP type I receptors [17] (Figure 1). However, some BMPs (BMP2, BMP11 and BMP16) activate Smad2 and Smad3 due to their binding to TGF-β receptors [20,21,22,23].

Once R-Smads become phosphorylated, they bind to the Co-Smad to form a complex, which translocate to the nucleus, where it regulates the transcription of target genes through binding to the Smad-binding elements (SBEs) or GC-rich sequences in the gene promoter region. The affinity and selectivity between the Smad complex and SBE motif is relatively low; thus, the interaction with various nuclear co-factors is necessary to ensure a specific and efficient activation of target genes [24]. The nuclear co-factors for the activated Smad complex include DNA binding partners and transcriptional co-activators or co-repressors. Different DNA binding partners result in differential regulation of target genes in a cellular context-dependent manner. Among the many DNA binding proteins that have demonstrated to interact with R-Smads in the nucleus are Runt-related transcription factor (Runx), O/E1-associated zinc-finger protein (OAZ) and Msh homeobox 1 (Msx1) [25]. Additionally, upon binding to DNA and to their transcriptional partners, Smads recruit also co-activators, such as p300, C/EBP-binding protein (CBP) and p300/CBP-associated factor (P/CAF), facilitating the initiation of transcription [26]. On the other hand, the recruitment of co-repressors, such as Ski and SnoN, by Smads is also well demonstrated [27]. 

Although the Smad-dependent pathway is the most studied BMP pathway, yet there are still uncertainties as to its mechanism of activation.

### 2.3. Non-Smad Pathways

In addition to the canonical Smad signalling pathway, BMPs activate also non-canonical pathways in a cell-type and cell-context specific manner resulting in tightly regulated cell-specific responses [28]. Several non-Smad pathways for BMPs have been identified; among these, mitogen-activated protein kinase (MAPK) pathway (p38, ERK and JNK) as well as phosphatidylinositol 3-kinase (PI3K)/AKT, PKC, Rho-GTPases, Lin-11/Isl-1/Mec-3 gene products kinase (LIM kinase) and others (Figure 1), which form a complex network of molecular signals regulating a multitude of processes throughout the body [29]. The specific mechanisms driving activation of these non-canonical pathways by BMP ligands are diverse and poorly understood. BMPR activation leads to MAPK activation via TGF-β-activated kinase (TAK) and TAK binding proteins (TABs) while p38 activation requires integrin-linked kinases [30]. BMP-induced p38 signalling has also been described to require recruitment of BMPRII into a signalling receptor complex (BISC) and depend on membrane microdomain association of signalling receptors [13]. AKT is activated by BMPRIA and IB in a PI3K dependent manner, without physical association between PI3K regulatory subunit (p85) and receptors but involving association of PI3K with tyrosine phosphorylated proteins [31]. Microdomains could also be critical for AKT activation [32]. Certainly, the means for setting the balance between Smad and non-Smad signalling are far from being fully characterized but evidence points to a spatiotemporal regulation as one of many potential determinants. 

Recently, BMP signalling was demonstrated to directly control either the expression or the processing of a cohort of microRNAs (miRNAs 21, 30b/c, 96, 143/145 and 302), during BMP-mediated cellular differentiation, in particular vascular smooth muscle cells differentiation, osteogenesis and neurogenesis [33]. Such regulation occurs via Smads, involving both transcriptional-dependent and independent pathways, with the exception of miRNA-30b/c for which a Smad-independent regulation has been described.

### 2.4. BMP Signalling Regulators

BMP signalling is extensively regulated by extracellular, intracellular and membrane modulators (Figure 1). 

Important modulators of the BMP signalling are the extracellular regulators. Soluble secreted proteins include BMP antagonists and other BMP-binding molecules that behave either potentiating or inhibiting BMP signalling by sequestering, transporting and solubilizing BMPs. Currently over 15 known antagonists have been classified into three subfamilies based on size of the cysteine knot: the Dan proteins family with an eight-membered ring; the Twisted gastrulation (Tsg) family with a nine-membered ring; and chordin, noggin, ventroptin, follistatin and FLRG-follistatin-related gene with ten-membered rings [11,34]. Membrane/matrix-associated proteins, including fibrillin, type IV collagen or BMP binding endothelial regulator (BMPER), also interact with some BMP ligands and modulate their activity [35].

BMP signalling is also regulated by co-receptors from various protein families and decoy receptors. Co-receptors typically bind BMPs and one or both receptors to enhance or inhibit signalling in specific tissues. Co-receptors that potentiate BMP signalling include the repulsive guidance molecules (RGM)/Dragon family, receptor tyrosine kinases (RTK) such as c-Kit and muscle-specific kinase (MuSK) [36] and the TGF-β/BMP type III receptors endoglin and betaglycan [15]. Other RTKs, such as TrkC and Ror2, have been also found to directly bind to BMP receptors, BMPRII and ALK6, respectively but rather than potentiate, interfere with BMP signalling [37,38]. Decoy receptors for BMPs negatively regulate BMP-induced signalling by competing for the binding of ligands to type I receptors. BMP and activin membrane bound inhibitor (BAMBI) is a decoy receptor resembling BMP type I receptor but lacking an intracellular kinase domain, being then able to sequester BMPs from active receptors [39].

Similar to TGF-β signalling, BMP receptors and downstream Smad proteins can be regulated by ubiquitin ligases. Smurf1, Smurf2 and NEDD4-2 can negatively regulate BMP signalling by different means; interacting with R-Smad and Smad4 and targeting them for degradation; promoting the association between BMP type I receptors and I-Smads to induce degradation of receptors; and interacting with I-Smads to induce their nuclear export [40]. Other ubiquitin ligases such as Arkadia, positively regulate BMP signalling by triggering inhibitory Smad6 for degradation [41]. At this level, it is important to mention the contribution of different de-ubiquitylating enzymes [42], for example USP4 that removes the mono ubiquitination of Smad4 thus promoting BMP signalling [43].

The intricacy of the signalling cascade amplifies as it descends downstream. Besides interacting with extracellular ligands, co-receptors and decoy receptors, BMP receptors can also bind several intracellular proteins, which act as signal transducers or regulators. Intracellular regulators of BMP signalling include microRNAs, I-Smad, phosphatases such as PP1 and PP2A that dephosphorylate the receptor and R-Smad and FK506-binding protein 1A (FKBP1A or FKBP12) that binds the GS domain of type I receptors to inhibit receptor internalization [13]. Casein kinase II beta (CK2 beta), the regulatory subunit of CK2, has also been reported to interact with type I receptors ALK1 and ALK3 enhancing or reducing, respectively, its signalling and function [44,45]. Furthermore, Dorpholz ad co-workers have described that IRS4 impairs BMP signalling by physical interaction with BMPRII, which subsequently results in enhanced ubiquitination of Smad1 targeting it for proteosomal degradation [46]. The BMPRII/IRS4 interaction serves as a platform for inhibition of Smad signalling and activation of the PI3K/AKT axis. Crosstalk with other signalling pathways, including not only PI3K/AKT but Wnt and Notch signalling [47,48], as well as MAPKs, is another important layer of control. These signalling pathways not only can be activated by BMP receptors as described before (non-Smad pathways) but can mediate indirect communication with BMP signalling. In the case of MAPKs, the interplay involves phosphorylation of Smad1 linker region that lead to positive and negative feedback loops [49,50].

In addition to those just listed, a subgroup of the TGF-β superfamily ligands, such as Xnr3, Lefty, BMP3 and BMP15, exerts antagonism of BMP activity via direct interaction with BMPs, although how this antagonism is exerted is unclear [11]. Likewise, ligand antagonism based on the affinity for type II receptors has also been reported, such that high affinity ligands (activin A), out-compete ligands that bind receptors with low-affinity (BMP2, BMP7) [51].

## 3. BMP and Liver Physiology

Recent evidence indicates that the liver is an important target of BMPs. The best-known effect of BMPs in liver physiology is undoubtedly the control of iron homeostasis. It has been described that in hepatocytes BMP6 activates hepcidin expression via binding to its receptors and co-receptor hemojuvelin (a member of RGM) and activation of the Smad canonical pathway. Liver-derived hepcidin is a master regulator of systemic iron balance as it controls the entry of dietary iron into the blood stream, iron recycling from the erythrocytes and iron storage in ferroportin. How this system is switched on and off is not completely understood. It is known that hepatic iron regulates BMP6 expression, mainly in non-parenchymal cells, by a still not well-determined mechanism. Importantly, circulating iron stimulates Smad pathway and hepcidin expression in a BMP6-independent manner. Again, molecular mechanisms behind these processes remain to be further delineated. In any event, these data evidence a critical role of BMP signalling in the physiological control of iron homeostasis [52,53].

Recently, a role for BMP9 as a hypoglycemic factor was postulated [54]. Authors in this work found that BMP9 expression in liver is downregulated in different models of insulin resistance in rats, while it is upregulated in response to an oral glucose challenge, suggesting that BMP9 could improve glucose homeostasis in diabetic and non-diabetic rodents. In liver, BMP9 inhibits glucose production and induces the expression of key enzymes of lipid metabolism [55]. Furthermore, recent data indicate that diabetic patients present lower circulating BMP9 levels than healthy humans. Thus, BMP9 levels negatively correlate with different parameters assessing glucose homeostasis, such as haemoglobin glycosylated, fast glucose blood levels and HOMA-IR [56]. BMP9 is not the only BMP ligand with a role in glucose homeostasis and other metabolic functions. BMP2 and BMP4 would also contribute to glucose homeostasis acting either systemically or on pancreatic cells [57,58,59,60]. Additionally, BMP4 and BMP7 would regulate brown adipocytes differentiation and function, with important consequences in thermogenesis, energy homeostasis or lipolysis [61]. These interesting functions of BMPs have been extensively reviewed by other authors [53]. In this review, we wish to focus on other equally interesting but less known functions of BMPs, that is, their role in regulation of liver regeneration and fibrosis.

## 4. BMP and Liver Regeneration

### 4.1. BMPs in Hepatocyte-Mediated Liver Regeneration

The liver has a remarkable regenerative capacity. In adult human, the liver is a mitotically quiescent organ with most hepatocytes in the G0 phase and mitotically inactive [62]. Under physiological conditions, mature hepatocytes and biliary epithelial cells maintain the balance between cell loss and cell gain by dividing themselves [63]. But following partial removal of tissue, the remaining populations of hepatocytes and biliary epithelial cells (also known as cholangiocytes) have the capacity to meet replacement demands of cellular loss accounting for the extraordinary regenerative capacity of the liver. Specifically, the regeneration of the liver can be more correctly defined as compensatory hyperplasia and hypertrophy as the remaining liver tissue expands to meet the metabolic needs of the organism but the expanding liver does not regain its original gross anatomical structure [64].

Few reports have linked BMP signalling to the regenerative process in the liver. Gene ontogeny analysis demonstrated that BMP family members were transcriptionally inhibited after 2/3 partial hepatectomy (PH) [65]. That is the case of BMP2 [66] and BMP4 [67], whose expression decreases transiently after PH. Consistent with this, during the regenerative process a decreased phospho-Smad1,5,8 levels are observed in the liver. Furthermore, ALK3^−/−^ mice display an augmented hepatocyte proliferation after PH and in the same line of evidence, adenovirus mediated expression of noggin increases restoration of liver mass and increases hepatocyte proliferation in vivo [67]. These results suggest that inhibition of ALK3 or BMP signalling could represent a therapeutic strategy to enhance proliferation and regeneration. Indeed, chemical inhibition of ALK3 using a LDN-193189 compound accelerates restoration of liver mass over time by increasing hepatocyte proliferation rate after PH, effects that were not observed with an antagonist of ALK2 [68]. Recently, we have described that BMP9 elicits antiproliferative effects on hepatocytes and its expression is also downregulated in response to PH in mice, with a minimum expression after 2 days and a slow recovery afterwards. Furthermore, injection of recombinant BMP9 leads to a marked decrease in proliferation and an aggravation of the liver damage after PH, with increased serum levels of lactate dehydrogenase (LDH), aspartate aminotransferase (AST) and alanine transferase (ALT) [69]. Together, these data suggest that BMP signalling negatively regulates hepatocyte proliferation and needs to be downregulated to allow hepatocyte replication after PH. Whether there is partial or total redundancy between these BMP ligands remains elusive.

Contrariwise, BMP7 has been reported to facilitate liver regeneration. Administration of recombinant BMP7 results in an increased regeneration as compared to control mice, concomitant with a reduced liver damage and with an increased hepatocyte proliferation. Interestingly, ALK3 expression was found in healthy liver and upregulated in regenerating liver. However, BMP7 was not expressed in hepatic tissue, in contrast, authors hypothesized that circulating BMP7, released from the kidney, targets the liver to promote regeneration, thus acting as a pro-regenerative endogenous hormone. Supporting this idea, both ALK3-Fc, a chimeric protein acting as a ligand trap for BMP7 and a BMP7 neutralizing antibody result in an impaired regenerative response [70]. These data are in agreement with those obtained in a liver regeneration model in zebrafish. Dominant negative BMP receptor results in a reduced liver mass 7 days after PH, indicating that BMP signalling is required for liver regeneration in zebrafish. Furthermore, BMP2 signalling regulates zebrafish hepatocyte proliferation in vitro [71]. 

Altogether, the scenario is far from simple, with various BMP ligands showing either similar or opposing effects. It is difficult to explain the differences found among these ligands in the regulation of the hepatic regenerative process, as they share receptors and the Smad downstream signal transducers. Certainly, our knowledge about the role of BMP signalling in liver regeneration is very limited, which does not help much to propose a solid hypothesis. Important basic aspects such as the target cell for each BMP ligand, which might differ among them, or the signalling pathways implicated in the elicited responses, which could include both Smad-dependent and independent pathways, are missing. Significant efforts are needed to help us clarify these aspects of BMP functionality.

It has also become clear nowadays that cell response to a single signal does strongly depends on other signals present in a particular moment that can alter the response [72]. In this sense, efforts should be made to delineate the signalling crosstalks operating in liver cells during the regenerative process because although much information is available about the role of individual players during liver regeneration, we do not know that much about redundancy, compensation and crosstalk phenomena between signals.

An example of crosstalk that we have recently described is that occurring between the RTKs Epidermal Growth Factor receptor (EGFR) and Met/Hepatocyte Growth Factor (HGF) and TGF-β pathways unveiled through the generation of a transgenic mouse model that specifically expresses in hepatocytes a truncated form of the human EGFR lacking the catalytic domain (∆EGFR) and therefore acting as a negative dominant mutant [73]. When submitted to PH, these mice presented a delayed hepatocyte proliferation, concomitant with an increased TGF-β expression and Smad3 phosphorylation and amplification of its cytostatic effects. These results suggest that EGFR pathway modulates TGF-β pathway in the context of liver regeneration. In spite of the mentioned alterations, an apparently full regenerative response was achieved in ∆EGFR mice, associated with higher levels of HGF and higher levels of phosphorylated Met in regenerating liver, suggesting a compensation phenomenon. Additional studies evidence specific non-overlapping and cooperative effects of these two RTKs signalling pathways during liver regeneration [74] required for a complete and efficient regenerative response. 

Whether BMP signalling interplays with these and other signals during the hepatic regeneration process is not known yet. In this regard, it is important to mention that crosstalk between BMPs and other signals does exist in other regenerative processes. For instance, a bidirectional regulation of expression appears to exist between HGF and BMP2, a crucial mediator in bone formation during fracture healing. Thus, in human osteoblasts HGF increases BMP2 production [75], while blocking Met signalling inhibits BMP2-induced HGF production and enhances BMP2-induced osteoblast differentiation [76], suggesting that a signalling balance between these pathways plays a role during osteoblast differentiation. Furthermore, HGF induces BMPR2 expression in mouse fibroblasts and muscle-derived mesenchymal cells, thus contributing to fracture repair [77], so the crosstalk is not restricted to osteoblasts and seems to involve not only regulation of ligand expression but also of specific receptors.

### 4.2. BMPS in Liver Regeneration during Chronic Liver Injury

Although the liver has a great regenerative capacity, there are situations where hepatocytes and/or cholangiocytes regenerative capacity is compromised, such as chronic injury states in which adult hepatocyte proliferation is inhibited or exhausted. In these situations, expansion of putative liver progenitor cells (LPCs) in the periportal area occurs to support or take over the regenerative process [78,79]. This phenomenon is also known as ductular reaction due to the fact that these cells often form duct-like structures and LPCs in rodent experimental models of liver injury are commonly known as oval cells because of its oval-shaped nuclei. The ductular reaction is a complex process still largely unknown. It involves activation of oval cells precursors; whose precise origin is still under debate. For many years, they were thought to come from the canal of Hearing in the biliary compartment but new theories are arising pointing also to so-called hybrid hepatocytes contributing to ductular reaction by phenotypic conversion or metaplasia at least in certain contexts [80]. The most general consensus now is that the degree and/or type of injury affects the composition of LPCs niche and determines their fate during the regenerative process. Expansion of LPCs is followed by migration into liver parenchyma and differentiation into hepatocytes or cholangiocytes to replenish cellular loss and re-establish liver function. Evidence suggests the involvement of BMP in the regulation of the multi-step process of the ductular reaction, then joining the broad group of cytokines, growth factors and hormones that have been shown to regulate this intricate process [81]. 

Some evidence points to BMP4 as key inducer of hepatocyte phenotype in LPC. Thus, BMP4 is able to induce both mid (albumin) and late (glucose-6-phosphatase and tyrosine aminotransferase (TAT)) phase hepatic markers in WB-F344 rat epithelial hepatic progenitor cells [82,83]. Further supporting this idea, BMP4/Smad1 axis has been described to contribute to hepatoblast terminal differentiation in vivo [84] and is required for hepatic specification of mouse embryonic stem cell-derived definitive endoderm [85] and regulates the expression of hepatic differentiation genes in hepatic stem cells via recruiting transcriptional co-activators p300 and CREB binding protein (CBP) [86]. Furthermore, BMP4 promotes differentiation of hepatocellular carcinoma (HCC) cancer stem cells [87]. On this basis, BMP2 and BMP4 have been widely used in in vitro protocols of generation of hepatic cells from induced pluripotent stem cells (iPS) and from embryonic stem cells (ESC) [88,89]. All these data suggest that BMP4 is involved in hepatocytic differentiation of liver progenitor and stem cells in different physiopathological contexts. Less data about the role of other BMPs in LPC-mediated liver regeneration is available. For example, Nakasutka et al. have reported a transient expression of BMP2 in oval like-cells after acute treatment with CCl_4_ [90], suggesting a role in regulation of oval cells in this model, although it should be noted that this is not a model of oval cell-mediated regeneration, so the relevance of this phenomenon for the outcome of the regenerative response upon CCl_4_ damage is under question. 

A key role for BMP signalling in LPC-mediated liver regeneration has been uncovered using a zebrafish model [91]. Interestingly, hepatic expression of BMP-related genes, such as Smad5, is upregulated during LPC-driven liver regeneration. Additionally, both inhibition of BMP using the BMP selective inhibitor DMH1 and overexpression of dominant negative bmpr1 resulted in sustained Notch signalling and a reduced hepatocyte nuclear factor-4α (HNF-4α) expression, being HNF-4α considered a key member of the complex regulatory network that defines and maintains the hepatocyte phenotype and an absolute requirement for hepatocyte differentiation and epithelial morphology [92,93]. These results were confirmed in Smad5 mutants and further supported by in vitro studies showing that BMP2 is able to induce hepatocyte markers such as glucose-6-phosphatase catalytic subunit, TAT and tryptophan 2,3-dioxygenase (TDO) in a murine oval cell line. Altogether, results evidence a role for BMP signalling in differentiation of HPC into hepatocytes via Smad5/Tbx2b. In addition to its effects on hepatocytic differentiation, BMP signalling controls proliferation of newly-generated biliary epithelial cells via Id2 [91]. 

**Figure 2:**
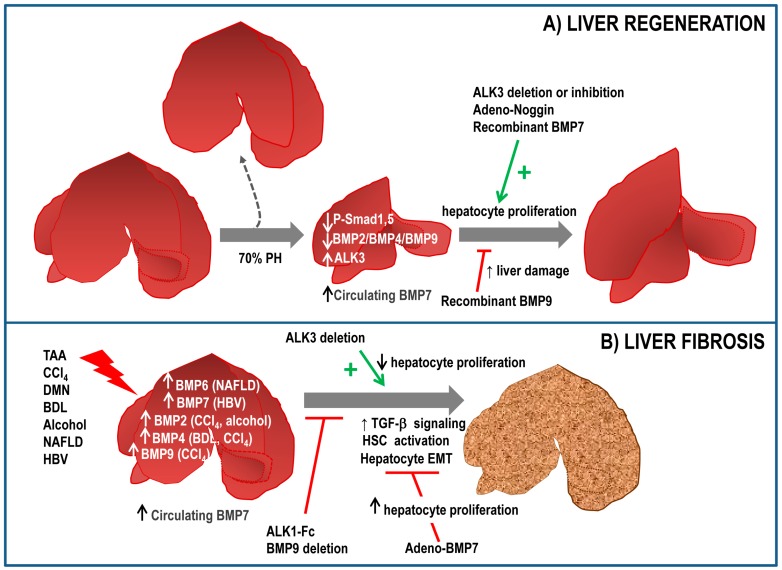
Role of BMP signalling in liver regeneration (**A**) and liver fibrosis (**B**). (**A**) Following a partial hepatectomy (PH), BMP signalling is reduced in liver, with a decreased expression of BMP2, BMP4 and BMP9 and a decrease in phospho-Smad1,5,8 (P-Smad1,5) levels. Different approaches show that BMP7, adenovirus-mediated expression of Noggin or ALK3 (BMP receptor type 1A) deletion/inhibition have a pro-regenerative effect in liver, whereas other BMP ligands such as BMP9, increase liver damage; (**B**) An upregulation of different BMP ligands has been observed in response to liver insults of different nature, such as bile duct ligation, CCl_4_ treatment and others. Accumulating data evidence a protective role for BMP7 in the context of liver fibrosis. Inhibition or deletion of BMP9 has a similar antifibrotic effect, while ALK3 inhibition favours liver fibrosis. The role of other BMPs is currently a matter of study. PH: partial hepatectomy; TAA: thioacetamide; DMN: dimethylnitrosamine; BDL: bile duct ligation; NAFLD: Non-Alcoholic Fatty Liver Disease; HBV: hepatitis B virus; EMT: epithelial to mesenchymal transition; HSC: hepatic stellate cells.

Recent data from our laboratory indicate that BMP9 also regulates LPC-mediated liver regeneration. Thus, expression of BMP9 and its receptors is decreased upon 3,5-diethoxycarbonyl-1,4-dihydrocollidine (DDC) treatment and absence of BMP9 results in an amplified oval cell expansion from the periportal regions followed by an improved liver regeneration in DDC-treated mice, supporting a role for BMP9 as a negative regulator of oval cell expansion and oval cell-mediated liver regeneration. These in vivo observations have been confirmed by in vitro studies using oval cell lines, in which BMP9 decreases oval cell number and increases apoptosis [94] (Figure 2A). These results broaden the spectrum of BMP9 actions in liver regeneration, including its cell target populations in liver.

## 5. BMPs and Liver Fibrosis

Liver fibrogenesis is a dynamic process that progressively leads to an excess in deposition of extracellular matrix (ECM) components, in an attempt to limit the consequences of chronic parenchymal injury [95]. This process is sustained and modulated by a crosstalk occurring between different hepatic cell populations, resident or recruited into chronically injured liver, that are involved in the synthesis and release of several mediators, including growth factors, cytokines, chemokines, adipokines, reactive oxygen species (ROS), vasoactive agents and plasma proteins. The cellular and molecular mechanisms of hepatic fibrogenesis have been extensively investigated using multiple complementary experimental animal model systems [96] and have led to the identification of different pro-fibrogenic mechanisms including chronic activation of the wound healing response; oxidative stress; a disarrangement of epithelial-mesenchymal interactions; epithelial to mesenchymal transition (EMT) of parenchymal cells; hepatocyte loss and chronic inflammation [95].

The major cell type responsible for cell repair appears to be the activated myofibroblast. The activation of quiescent hepatic stellate cells (HSC) is a pivotal feature of liver fibrosis pathogenesis, as these cells suffer profound changes in behaviour, including proliferation, chemotaxis, contractility, altered matrix degradation and production of fibrous scar, retinoid loss and inflammatory signalling [95,97,98,99]. TGF-β is a key player during liver fibrosis. It is known that TGF-β triggers the activation of HSCs, regulates ECM synthesis, provides cell contraction and migration capacity, induces apoptosis in hepatocytes and its signalling induces oxidative stress and consequently inflammation [100]. 

In contrast with this well-established role for TGF-β in hepatic fibrosis, the possible involvement of BMPs in this process has only recently been suggested. Most of the knowledge we have about the role of BMPs during the fibrogenetic process comes from kidney fibrosis studies, which have shown a protective antifibrotic role for BMP7 [101]. BMP7 inhibits or reverses fibrosis in experimental models of chronic kidney disease, mainly by antagonizing TGF-β–induced EMT [102,103,104]. 

In the liver, adenovirus mediated ectopic expression of BMP7 suppresses thioacetamide (TAA)-induced hepatic fibrosis in rats, reducing the expression of α-smooth muscle actin (αSMA), type I collagen and hydroxyproline content. BMP7 overexpression in primary HSC reduced the expression of collagen and αSMA, being this effect associated with an increased phospho-Smad1/5 [105]. Oral administration of recombinant adeno-associated virus carrying BMP7 in mice results in a long-term ectopic expression of BMP7 in the gastrointestinal mucosa and in an increased circulating BMP7 concentration. In these conditions, CCl_4_-induced hepatic fibrosis and HSC activation are blocked, measured by Masson trichrome staining, hepatic hydroxyproline content, serum hyaluronic acid concentration and by αSMA staining. Interestingly, ectopic expression of BMP7 promotes parenchymal cell proliferation, specifically hepatocytes [106]. These results indicated that BMP7 promotes hepatocyte regeneration and inhibits liver fibrosis, which are in agreement with its role as a pro-regenerative factor in the model of PH [70] and its protective effects in hepatocytes upon alcohol injury [107]. Additional evidence pointing to BMP7 as a pivotal factor regulating hepatic fibrogenesis comes from analysis of antifibrotic drugs, such as butyledenepthalide [108]; herbal compound 861 [109] and the Danshao Huaxian capsule [110]. In all cases, the therapeutic effect against hepatic fibrosis was associated with an up-regulation of BMP7.

Abundant in vitro and in vivo evidence supports the concept that the antifibrotic actions of BMP7 are due to its ability to counteract TGF-β expression, signalling and actions. In a model of hepato-schistosomiasis-induced fibrosis, recombinant BMP7 reduced histopathological features of fibrotic lesion and diminished TGF-β1 and αSMA expression in liver, results that again suggest that BMP7-antifibrotic effects might be dependent on its action on the TGF-β pathway [111]. In the classic CCl_4_-induced liver fibrosis mouse model, BMP7 antifibrotic effects have been proposed to rely on its crosstalk with EGFR and TGF-β1, by suppressing their expression and inhibiting activation of EGFR [112] and abolishing the accumulation of hepatocyte-derived fibroblasts via EMT [112]. Likewise, BMP7 reduces TGF-β levels in liver in a rat model of liver fibrosis mediated by injection of porcine serum. Interestingly, BMP7 therapeutic effects were observed both when administered before fibrosis development (prevention) and once fibrosis had already developed (treatment), widening its potential clinical applications [113].

How this BMP7/TGF-β crosstalk operates from a mechanistic point of view remains elusive. In renal fibrosis, where research is more advanced, various mechanisms have been proposed. BMP7 can counteracts TGF-β-induced EMT by reinduction of E-cadherin via a Smad-dependent pathway [114], by reducing nuclear translocation of Smad3 and blocking Smad3-dependent transcription in a Smad5-dependent but non-canonical (ERK1/2, p38, JNK)-independent pathway [115]. While these authors claim that BMP7 does not utilize the TGF-β transcriptional repressors Ski or SnoN, others [116] reported that BMP7 reduces Smad3 binding to its consensus SBE through regulation of the transcriptional repressor SnoN. The hypothesis of a Smad-dependent pathway rather than a non-canonical pathway-mediated effect of BMP7 is also supported by other studies [117], even in pulmonary fibrosis where BMP7 would act through a Smad1/5/8-Id2 and Id3 signalling pathway [105,118].

Despite these observations, the antifibrotic effect of BMP7 is still controversial due to the fact that in patients with cirrhosis and chronic hepatitis B as well as children with biliary atresia, an increase in BMP7 expression in hepatocytes and/or increased levels of BMP7 in serum have been found [119] and at least in some cases it was associated with higher disease severity [119]. Furthermore, results from Tacke et al. [119] using human telomerase reverse transcriptase (hTERT) immortalized human HSCs in vitro showed that infection with adenoviruses encoding BMP7 increased HSC proliferation. One could hypothesize that the rise on BMP7 during liver disease is an adaptive response to liver damage and fibrosis, so that the increased BMP7 tries to counteract profibrotic effect of TGF-β in the fibrotic liver. It is plausible that the increased BMP7 may not be sufficient to neutralize TGF-β. Supporting this, an interesting work from Bi et al. showed that in order to observe a counteractive action of BMP7 on TGF-β mediated EMT, TGF-β/BMP7 ratio must be at least 1/10 [120]. Hence, the fibrogenic process continues despite the increase in antifibrotic BMP7. Further work is necessary to confirm this hypothesis and to explain other unsolved matters, such as the opposing pro-fibrogenic [119] and anti-fibrogenic [105,108,112] actions described for BMP7 in HSCs. 

There are other BMP ligands involved in organ fibrosis, particularly in liver fibrosis. Thereby, BMP4 exerts a profibrotic role in liver during bile duct ligation-induced liver fibrosis in rats through activation of Smad1 and ERK-MAPKs in HSCs [121]. Moreover, upregulation of BMP4 expression during liver injury has been described both in animal experimental models and human patients. For instance, ERK1 upregulation during dimethynitrosamine induced-hepatic fibrosis is associated with induction of BMP4, together with TGF-β and PDGF, which was inhibited with AdshERK1 treatment that reverses EMT [122]. BMP4 produced by liver sinusoidal endothelial cells (LSEC) increases Hepatitis C virus replication in hepatocytes. A negative regulation of BMP4 by vascular endothelial growth factor-A (VEGF-A) signalling via a VEGFR2/p38-MAPK-dependent pathway was also reported [123]. The exact cell population producing BMP4 is not clear, as other reports indicate that BMP4 is expressed in hepatocytes and HSC [113,121]. Importantly, a genome-wide gene expression profile established during hepatitis B virus-induced HCC model pointed to BMP7 and BMP4 as candidates for common regulators of genes involved in the non-tumour to tumour-transition. In the same work, authors demonstrated that BMP4 and BMP7 were upregulated in patients with cirrhosis, HCC and cholangiocarcinoma and the increase correlated with the progression of cancer [124]. But once again, controversy is served. Nakatsuka et al. observed that BMP2 along with BMP4 is transiently expressed during early stages of CCl_4_-induced fibrosis in rats [90] but when they explored the relevance of BMP signalling in this context using conditional KO mice for ALK3^−/−^, an impaired hepatocyte proliferation in the wound healing response to acute CCl_4_ injury was observed, results that suggest a role for BMP signalling in amelioration of acute liver injury [125]. It might well be that the role played by BMP signalling in acute versus chronic liver injury is different. 

Although evidence in the literature indicates that BMP2 is involved in fibrotic process in different organs, including renal, lung, pancreatic and cardiac fibrosis, its potential role in liver fibrosis is so far only based on its induction during CCl_4_-induced fibrosis in rats [90] and chronic alcohol exposure in mice [126]. In the alcohol-induced injury model authors proved that activation of the receptor and the Smad1,5 pathway was however inhibited. We could hypothesize that this is another example of negative regulation between BMP and TGF-β signalling, such that BMP2 signalling might be abrogated by TGF-β signalling that is augmented upon alcohol exposure [126]. Lastly, in vitro studies in cultured HSC indicate that both BMP2 and BMP4 increase αSMA expression more potently than TGF-β, suggesting a role of transdifferentiation in myofibroblasts [127]. 

A recent work by Ardnt et al. describes that BMP6 is upregulated in Non-Alcoholic Fatty Liver Disease (NAFLD) but not in other types of liver injury (i.e. alcoholic liver disease, chronic viral hepatitis B and C cirrhosis). Interestingly, BMP2 and BMP4 were also upregulated in NAFLD but only BMP6 expression increased in an in vitro model of hepatocyte steatosis. More importantly, BMP6^−/−^ mice displayed more hepatic inflammation and fibrosis when subjected to methionine choline-deficient and high-fat diets, two models of Non-Alcoholic Steatohepatitis (NASH). Further analysis also reveals that recombinant BMP6 counteracts HSC activation and decreases pro-inflammatory and pro-fibrogenic gene expression once HSC are activated. All these data strongly suggest that BMP6 could have an antifibrotic role in NAFLD [128].

Our recent work has demonstrated that BMP9 is upregulated in experimental models of liver fibrosis and more importantly, loss of BMP9 signalling (BMP9-KO and ALK1-Fc mice) results in a significant decrease in the CCl_4_-induced fibrotic process, as evidenced by lower Col1a1 expression and decreased collagen fibres in the tissue and lower αSMA protein levels in the liver [69]. We have also observed that BMP9-KO mice show evidence of an amelioration in the degree of fibrosis and liver damage based on the decreased expression of fibrogenic markers, lower accumulation of collagen and lower levels of ALT, AST in serum after a DDC diet, a model of cholestatic liver injury [94] further supporting a pro-fibrogenic role for BMP9 in the liver. Additional indirect evidence points in the same direction. Li et al. have described that BMP9 via ALK1/2 and Smad1 activation triggers EMT in liver cancer cells [129]. Considering the tight connection between EMT and liver fibrosis and its relevance for HCC development, it is easy to speculate on the role of BMP9 as a profibrotic factor during the preneoplastic stages of liver cancer. Recently, it has been published that BMP9 induces the expression of collagen, fibronectin and connective tissue growth factor (CTGF) in cultured mouse fibroblasts [130]. Moreover, BMP9-KO mice present a defect in matrix deposition [131], which is a key process in fibrogenesis, serving therefore as an indirect evidence of a role for BMP9 in this process (Figure 2B).

For a complete picture of the relevance of BMP signalling in liver fibrosis we ought to take into account the role of BMP signalling modulators (Table 1). 

Endoglin is a transmembrane protein that functions as a co-receptor for TGF-β/BMP signalling. Endoglin binds TGF-β1 and TGF-β3 with high affinity and also activin, BMP2 and BMP7. In all cases this binding requires the presence of the signalling receptor [146,147]. Recently, BMP9 has been shown to directly interact with endoglin and interestingly, the affinity of this interaction is comparable to BMP9-ALK1 interaction and is independent of the presence of other receptors [148]. Although in other systems both pro- and antifibrotic actions for endoglin have been described, in hepatic fibrosis accumulating evidence points towards a profibrotic role for this protein. Both circulating and intrahepatic levels of endoglin are increased in patients with chronic hepatitis C and are associated with fibrosis progression. Intrahepatically, expression was found in HSC and portal and septal myofibroblasts [132]. Furthermore, endoglin expression increases in HSC in two different models of liver fibrosis (bile duct ligation and CCl_4_ administration) [133]; and in HSC undergoing transdifferentiation in vitro [133,134].

How endoglin mechanistically influences hepatic fibrogenesis is a matter of extensive study. In endothelial cells, where endoglin is highly expressed, endoglin shifts TGF-β from ALK5/Smad2-3 to ALK1/Smad1 pathway. Although there is no full consensus [149], most studies agree that endoglin potentiates Smad1 pathway in other cell types as well. This is the case of HSC [133,150]. To understand the complexity of endoglin function during liver fibrogenesis is necessary to go beyond the TGF-β signalling modulation. Undoubtedly, endoglin impacts on TGF-β signalling contributing to its pro-fibrogenic effect but endoglin effects on BMP signalling must be also taken in consideration. Thus, endoglin increases BMP7-mediated transcriptional activity in myoblasts [151]. Since both endoglin and BMP7 are expressed in HSC, it would be interesting to know if a similar effect is accomplished in this population during liver fibrosis. Endoglin is known to interact with most type I and type II receptors and of those, ALK2, 3 and 6 and BMPR2 and ActR2 are binding receptor for BMPs, therefore a possible interaction between endoglin and other BMP ligands –apart from the already described BMP2, BMP7 and BMP9- could be hypothesized. Particularly interesting is the case of the axis BMP9/endoglin. As mentioned before, endoglin binds BMP9 with high affinity in the absence of the signalling receptors [148,152,153], it modulates positively BMP9-triggered signalling [154] and in some cell types, such as endothelial cells, it is required for BMP9-mediated gene regulation [155,156]. In light of our recent findings on the BMP9 role in liver fibrogenesis [69], it is conceivable that endoglin contribution to this process is related to BMP9. Further studies to solve this question are warranted. 

Connective tissue growth factor (CTGF) is a protein that belongs to the CCN family. It has been shown to interfere with BMP/TGF-β signalling networks [157]. CTGF binds to both BMP and TGF-β, antagonizing BMP activity by preventing binding to its receptor while having the opposite effect, enhancement of receptor binding, on TGF-β, being these actions involved in fibrosis [135,136]. Importantly, CTGF is expressed in hepatocytes upon liver injury [158,159]. Nevertheless, there is some controversy in the literature about the precise effect of CTGF on BMP signalling in the context of liver fibrosis, since contrary to the CTGF trapping effects on BMP4 and BMP7, other authors did not find evidence for an interaction of CTGF with BMP7 in hepatocytes [137]. Whether CTGF is capable of modulating other BMP ligands is not known and how this modulation would impact on liver fibrosis is still an unexplored question.

Gremlin is a glycoprotein that belongs to the Dan family, composed of 9 members. It binds to BMP2, BMP4 and BMP7 with high affinity and interacts with other TGF-β family members. Through its binding to the BMPs, gremlin blocks the downstream signalling, therefore acting as a BMP antagonist. Interestingly, upregulation of gremlin was observed in animal models of liver fibrosis and in cirrhotic patients [138,139,140] and it is expressed in fully activated HSC, which suggest its involvement in the process of activation into myofibroblasts. Indeed, Zhang et al. found a direct correlation between gremlin expression in HSCs and expression of fibrosis markers, αSMA, Col1a, TGF-β, proposing an important role for gremlin in HSC activation via upregulation of TGF-β and likely inhibiting BMP7 protective signals [140]. At the same time, BMP7 positively regulates gremlin expression while suppresses TGF-β expression [139]. These data evidence cross-regulatory loops between these molecules during liver fibrosis. More studies are needed to clarify how these regulatory loops work in this context and their effects on liver fibrosis outcome.

Direct and indirect evidence has linked other members of the Dan family, such as sclerostin and uterine sensitization-associated gene-1 (USAG1), which act also as BMP antagonists, with kidney disease but its involvement in liver fibrosis is poorly understood. It is worth mentioning that several reports have identified altered sclerostin expression in serum and/or liver of patients with NAFLD and cholestatic disease [160,161]. These reports focus on the importance of sclerostin upregulation in the context of an organ crosstalk between the liver and the bone but the consequences of increased hepatic expression of sclerotin in the liver itself have not yet been analysed.

Kielin/chordin-like protein (KCP) belongs to the chordin family whose members possess in their structure a cysteine rich (CR) domain containing a CXXCXC and CCXXC motif, a protein module for the binding and regulation of BMPs [162]. Specifically, KCP presents 18 CR motifs that interact with TGF-β/BMP family members. Whereas chordin, the prototype member of this family, binds to BMPs and blocks their interaction with their receptors, KCP exerts the opposite action, enhancing BMP-BMPR interaction resulting in an increased signalling [163]. On the contrary, KCP inhibits TGF-β/activin signalling [164]. In liver of aged mice or in mice fed with a high fat diet to induce NAFLD, loss of KCP promoted hepatic steatosis and fibrosis whereas expression of KCP transgene was protective [141]. These effects were mainly due to the blockage of TGF-β signalling rather than an effect on BMP signalling, contrariwise, in renal fibrosis KCP was able to modulate both TGF-β and BMP signalling. The remarkable effect of KCP on liver fibrosis deserve further analysis, it would be particularly interesting to analyse the contribution of BMP-modulation on KCP effects and how these data may translate into human disease.

Follistatin is a secreted glycoprotein that interacts with high affinity with activin and with lower affinity with other TGF-β/BMP ligands, such as BMP4, BMP5, BMP6, BMP7, BMP15, GDF9 and TGF-β3 [165]. The role of follistatin in fibrosis has been extensively reviewed before [165,166]. With respect to liver disease, evidence indicates that follistatin is a key modulator of liver function, mainly as a regulator of activin. Follistatin administration or overexpression by different means promoted DNA synthesis and liver growth and accelerated liver regeneration after PH. Importantly, follistatin levels are increased 24–48 h after PH [167]. Furthermore, small hepatocytes, a subpopulation of hepatocytes that are considered as “committed progenitor cells” require follistatin to proliferate [168]. Administration of follistatin in rats reduces liver damage in the CCl_4_-mediated liver fibrosis model [142] and in a model of isquemia–reperfusion injury [143]. Whether this hepatoprotective effect is also operative in human disease is not known. Increased levels of follistatin are found in liver pathological conditions, including Alcoholic Liver Disease (ALD), NAFLD, NASH and cirrhosis [161,169,170] and it is tempting to hypothesize that it could function as a protective mechanism trying to counterbalance the profibrogenic actions of activin. In the context of chronic liver disease, it would be important to analyse whether follistatin could evolve its protective action also controlling profibrotic BMP signalling or favouring BMP antifibrotic actions.

Bambi is a pseudoreceptor that was identified for its capacity to interfere with both BMP and TGF-β signalling, as it interferes with the formation of a functional receptor complex [39]. Bambi is expressed in liver [144], specifically in hepatocytes, cholangiocytes and HSC. Interestingly, Bambi expression diminishes in HSC upon activation [171]. Bambi downregulation has been described in different models of liver disease and this downregulation is thought to be achieved by a variety of mechanisms. For example, adiponectin controls the expression of Bambi, particularly in HSC. In the context fatty liver, circulating levels of adiponectin are reduced, which could explain the reduced levels of Bambi in the liver of patients with NAFLD. Interestingly, the decrease in Bambi levels is even more pronounced in NASH patients [144]. In quiescent HSCs, Toll like Receptor 4 (TLR4) activation downregulates Bambi and sensitizes HSCs to TGF-β–profibrotic signals [145]. Subsequent studies have described the same TLR4-dependent downregulation of Bambi behind the hepatic fibrogenic process triggered by accumulation of free cholesterol in HSC [172,173,174]. Of note, analysis of the involvement of Bambi in fibrosis has been essentially focused on the effects of Bambi on the TGF-β pathway. However, it is well established that BMP controls Bambi expression [39,175] and that Bambi modulates BMP signalling as well, although the exact nature of this modulation appears to be dependent of the cellular context [39,176], so the BMP/Bambi regulatory circuit might have some relevance in liver fibrosis. 

Other extracellular regulators of the BMP signalling such as noggin, chordin, among others, have been shown to be regulated in pulmonary fibrosis [177] but its involvement in liver fibrosis has not been systemically studied.

In summary, if we focus on HSC as major target cell for BMPs in liver fibrosis, evidence points to a scenario in which HSC transdifferentiation is modulated by different BMP ligands. BMP2 and BMP4 would act as pro-fibrogenic factors potentiating HSC transdifferentiation while BMP6 and BMP7 would impair this process therefore acting as anti-fibrogenic factors [105,112,121,127,128], although some debate exists about BMP7 precise role since it could also stimulate HSC proliferation [119]. On its side, BMP9 expression in HSCs increases during the transdifferentiation process, being able to modulate both HSC proliferation and migration [69]. Since endoglin follows the same pattern of expression (increased expression during the HSC to myofibroblast transition) [132,133,134] together with the fact that BMP9 and endoglin bind with strong affinity [148,152,153], it is conceivable to speculate that these two events are related. Based on this, analysis of the role of endoglin in BMP9 profibrotic effects in liver could be important. On this respect, it is also necessary to keep in mind that endoglin could modulate the actions of other BMP ligands, such as BMP2 and BMP7 [146,147]. We should not ignore either that activated HSCs show an altered expression pattern of BMPs modulators, specifically, high levels of gremlin and endoglin and lower levels of Bambi, as compared to normal HSC [133,134,140,171]. How the altered expression of these modulators affects BMPs signalling and final cell response is not known but we could hypothesize on an inhibition of BMP antifibrotic effects and promotion of profibrotic signals. In any case, a more systematic approach would be necessary to draw a complete picture about the actions of the different BMP ligands on HSC and hepatic myofibroblast, approach that should ideally include analysis of the contribution of Smad and non-Smad signalling branches and the mechanisms driving their effects.

### Perspective on BMP Signalling as Therapeutic Target in Liver Fibrosis

Significantly, despite the fact that BMP signalling dysregulation has been associated with many pathologies, the only FDA approved use of BMPs at present is that of recombinant ligands delivered in open fractures, vertebral fusion and maxillofacial bone augmentation [178]. 

Recognizing that much work is needed to completely characterize the function of specific BMPs in liver fibrosis associated with chronic liver injury of different aetiology, evidence gathered in this review could support approaches aimed at blocking BMP2/4 and BMP9 signalling or enhancing BMP7 and BMP6 (in NAFLD-associated fibrosis) as potential strategies that would ultimately lead to decrease the fibrotic process in the liver. Indeed, some of these ligands and specific extracellular regulators are already in clinical trials for their regulatory role in fibrosis in other tissues or in additional pathological contexts. Thus, it is worth mentioning that dalentercept, a ligand trap molecule for BMP9 and BMP10, is in clinical trial for its anti-angiogenic activity in solid tumours, specifically in HCC (NCT02024087). Evidence supporting the benefits of BMP9 inhibition in treatment of fibrosis is strong but certainly more pre-clinical data are required to put forward this molecule as an antifibrotic agent in the liver. TRC105, a monoclonal antibody that binds endoglin, is being analysed in more than 20 clinical trials as an anti-angiogenic drug in different tumour types but once again, a deeper knowledge on the exact function of endoglin in liver fibrosis is needed before taking a step forward. Pharmacological strategies designed to inhibit gremlin have been tested in different disease models. An antibody against gremlin is already developed and it might be an interesting strategy to treat liver fibrosis [179]. CTGF is also worth consideration since its down regulation would result in a shift in TGF-β/BMP7 balance towards an antifibrotic signalling. Preclinical data are indeed encouraging [180] and besides, a clinical trial for the use of a human monoclonal antibody targeting CTGF in idiopathic pulmonary fibrosis is being carried out (NCT00074698). Hopefully, results of this study will give us more clues about a possible use of similar approaches in liver fibrosis. Although BMP7 could be a promising tool in antifibrotic therapy in liver and different BMP7 delivery systems have been tested in different experimental models of fibrosis, there is no information about completed or ongoing clinical trials actually examining this possibility [181]. On this respect, THR123, an ALK3 stimulating peptide, has been shown to reverse both acute and chronic renal injury [182], proving that the use of engineered BMP ligands could be a suitable strategy to follow in fibrosis therapy. In any case, although holding promise, we have to keep in mind that BMP family members play critical roles in tissue homeostasis, therefore the challenge is to find an approach that specifically inhibits hepatic fibrosis progression without compromising the other BMP functions.

## 6. Concluding Remarks

In conclusion, a role for BMP signalling in the regulation of liver regeneration and liver fibrosis in different pathological situations seems unquestionable today. However, the scenario is complex and sometimes confusing. Important aspects including signalling mechanisms behind BMPs actions, specific target cell populations, redundancy, cross-regulatory circuits involving BMP/TGF-β modulators and crosstalk with other signals/pathways, must be clarified if we aim to understand the real relevance of these ligands in liver pathology and the real promise as therapeutic targets in specific pathological contexts. We are just at the beginning of a long path, quite challenging but certainly exciting. We hope this review serves to stimulate researchers moving in this direction.

## Figures and Tables

**Table 1:** Role of BMP signalling modulators in liver fibrosis. CTGF: Connective tissue growth factor; KPC: Kielin/chordin-like protein.

	Potential Effect on Liver Fibrosis	Regulatory Effect on BMP Signaling in Liver Fibrosis	References
**Endoglin**	Profibrotic	Not determined. Indirect data would support a stimulatory effect on BMP signaling	[132,133,134]
**CTGF**	Profibrotic	CTGF does not interfere with BMP7 signaling BMP7 inhibits CTGF expression	[135,136,137]
**Gremlin**	Profibrotic	It inhibits BMP7 antifibrotic signaling	[138,139,140]
**KCP**	Antifibrotic	Not determined	[141]
**Follitastin**	Antifibrotic	Not determined	[142,143]
**Bambi**	Antifibrotic	Not determined	[144,145]

## Abbreviations

BMP	Bone Morphogenetic Proteins
GDF	Growth Differentiation Factor
SBE	Smad-binding elements
MAPK	Mitogen-activated protein kinase
PI3K	Phosphatidylinositol 3-kinase
RGM	Repulsive guidance molecules
RTK	Receptor tyrosine kinases
PH	Partial hepatectomy
LPC	Liver progenitor cell
DDC	3,5-Diethoxycarbonyl-1,4-dihydrocollidine
EMT	Epithelial to mesenchymal transition
HSC	Hepatic stellate cells
αSMA	α-Smooth muscle actin
NAFLD	Non-Alcoholic Fatty Liver Disease
NASH	Non-alcoholic steatohepatitis
KPC	Kielin/chordin-like protein
CTGF	Connective tissue growth factor
USAG1	Uterine sensitization-associated gene-1
TLR4	Toll like Receptor 4
